# Production of a Self-Aligned Scaffold, Free of Exogenous Material, from Dermal Fibroblasts Using the Self-Assembly Technique

**DOI:** 10.1155/2016/5397319

**Published:** 2016-03-09

**Authors:** Stéphane Chabaud, Stéphane Bolduc

**Affiliations:** ^1^Centre LOEX de l'Université Laval, Génie Tissulaire et Médecine Régénératrice, LOEX du Centre de Recherche FRQS du Centre de Recherche de CHU de Québec, Axe Médecine Régénératrice, Aile-R Centre Hospitalier Affilié Universitaire de Québec, 1401 18e rue, Québec, QC, Canada G1J 1Z4; ^2^Département de Chirurgie, Faculté de Médecine, Université Laval, Québec, QC, Canada G1K 7P4

## Abstract

Many pathologies of skin, especially ageing and cancer, involve modifications in the matrix alignment. Such tissue reorganization could have impact on cell behaviour and/or more global biological processes. Tissue engineering provides accurate study model by mimicking the skin and it allows the construction of versatile tridimensional models using human cells. It also avoids the use of animals, which gave sometimes nontranslatable results. Among the various techniques existing, the self-assembly method allows production of a near native skin, free of exogenous material. After cultivating human dermal fibroblasts in the presence of ascorbate during two weeks, a reseeding of these cells takes place after elevation of the resulting stroma on a permeable ring and culture pursued for another two weeks. This protocol induces a clear realignment of matrix fibres and cells parallel to the horizon. The thickness of this stretched reconstructed tissue is reduced compared to the stroma produced by the standard technique. Cell count is also reduced. In conclusion, a new, easy, and inexpensive method to produce aligned tissue free of exogenous material could be used for fundamental research applications in dermatology.

## 1. Introduction

Self-assembly technique allows the production of scaffolds free of exogenous material from various sources of mesenchymal cells such as dermal fibroblasts [1, 2], corneal keratocytes [3], bladder mesenchymal cells [4], and adipose-derived stromal cells [5]. The mesenchymal cells produce, secrete, and assemble the extracellular matrix (ECM) themselves. As this method reproduces a near physiological matrix environment, the reconstructed engineered tissues could be used to mimic some pathologies (e.g., wound healing and scars [6], scleroderma [7], psoriasis [8], melanoma [9], or dermal aspects of amyotrophic lateral sclerosis [10]).

Orientation of the cells or the matrix fibres plays a key role by modulating the extracellular matrix deposition [11, 12] and remodelling [13], the cell migration [13], differentiation [12, 14] and proliferation [15], and also the mechanical properties of the tissue in which the cells are embedded [16–19]. Pathologies which involve modifications in the alignment of the matrix are numerous, for example, cancers [20], cutis laxa [21], the eye sclera in diabetes [22], or glaucoma [23]. Ageing also involves a change in collagen fibre orientation [24–26] and botulinum toxin-A treatment improves collagen fibre alignment [27]. Various techniques have been used in the past to produce aligned matrix. Several of these techniques involve a biocompatible synthetic scaffold [28, 29] or require sophisticated materials [30] such as micropatterned [31–33] or chemically modified surfaces [34, 35].

With the expansion of lifespan in human populations, two medical conditions raise an interest for variation of composition and structures of the skin: the ageing and cancer. Moreover, the photoageing, induced by the exposition of unprotected skin to the sun, attracts the attention of a lot of dermatologists. Resident fibroblasts in aged dermis present elevated levels of matrix metalloproteinases (MMPs) inducing remodelling and thinning of the skin with flat aspect which results from a loss of papillary structure in the epidermis-dermis junction [36, 37]. It was also found that an alignment of collagen fibres in the neighbourhood of precancerous lesions could influence the behaviour of the nascent tumour. The remodelling of the ECM of the tumour leading to realignment and its stiffening is modulated through integrins and Rho-Rho associated protein kinase (ROCK) by several molecules such as MMPs, syndecan-1, lysyl-oxidase (LOX), yes-associated protein (YAP), caveolin-1, transforming growth factor-beta (TGF-beta), fibroblast activation protein (FAP), and platelet-derived growth factor (PDGF), but also by physical conditions such as stiffness, strain, or interstitial flow [38]. Cancer myofibroblasts, by depositing and contracting the ECM, contribute also to modifying this microenvironment [36]. During the growth of the tumour mass, hypoxia induced by the tumour promotes the remodelling and stiffening of the matrix [39]. The alignment of the matrix plays a key role in the evolution of the tumour [40] and especially in its invasive and metastatic potential [41–43].

The organization of the matrix, especially collagen fibre orientation, and its consequences on cells are investigated using animal models or tissue engineering techniques such as the one described above. Cells react to stretch or mechanical load by realigning the extracellular matrix [44–47]. This reorganization of collagen fibres allows adaptation to new physiological conditions [48]. This feature could be used to produce tissues with aligned collagen fibres using the self-assembly technique. This study proposes a new technique to produce a tissue, free of exogenous materials and relatively inexpensive, where matrix fibres and cells are horizontally aligned for use in clinical applications (if tissues need to be more structured, such as corneal stroma) or fundamental researches (reproduce dermis from cancerous or ageing people). Except for a plastic ring to elevate the construct, no special material is required.

## 2. Materials and Methods

### 2.1. Cell Culture

Ethical considerations: all procedures involving patients were conducted according to the Helsinki Declaration and were approved by the local Research Ethical Committee. Informed consent of donors was obtained for each specimen. Human dermal fibroblasts (Fb) were used as mesenchymal cells to produce the stroma. Skin biopsies were collected from healthy donors during a plastic surgery. The skin specimen was washed in phosphate buffered saline (PBS) containing 100 U/mL penicillin (Sigma, Oakville, Canada), 25 mg/mL gentamicin (Schering, Pointe-Claire, Canada), and 0.5 mg/mL Fungizone (Bristol-Myers Squibb, Montreal, Canada); then it was cut in small pieces of 1 mm. It was then incubated at 4°C overnight with 10 mL of a solution containing 500 mg/mL thermolysin (Sigma) in HEPES buffer with 1 mM CaCl_2_, pH 7.4. The next day, the dermis was manually separated from the epidermis and incubated for 3 hours at 37°C with agitation in a solution of 0.125 U/mL collagenase H (Roche Diagnostics Canada, Montreal, Canada) diluted in Dulbecco-Vogt modification of Eagle's medium (DMEM, Invitrogen, Burlington, Canada) containing 10% foetal bovine serum (Hyclone, Logan, UT), 100 U/mL penicillin, and 25 mg/mL gentamicin (Fb medium). Fb collected by centrifugation were then seeded in culture flask at 6 × 10^4^ cells/cm^2^ in Fb medium and cultivated at 37°C in a humidified 8% CO_2_ atmosphere. Medium was exchanged three times a week. Three cell populations were used.

### 2.2. Engineered Stroma Production

**Figure 1:**
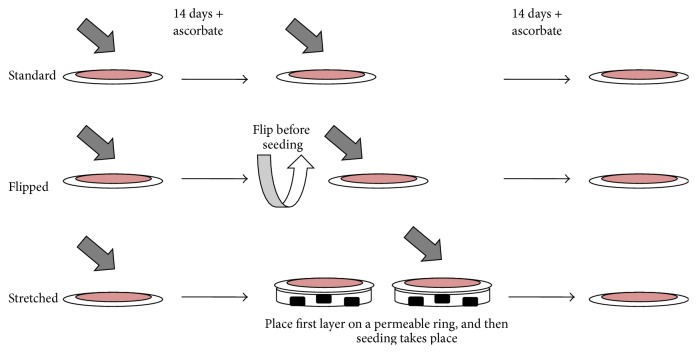
Experimental design of the cell culture part of the study. Fibroblasts were seeded as a confluent layer and cultivated with ascorbate for 14 days. A reseeding of fibroblasts took place: without modification for standard condition, with a flip of the matrix sheet before the seeding for the “flipped” condition, and with the elevation of the matrix sheet on a permeable ring of plastic for the “stretched” condition. All the cultures were pursued for 14 days.

**Figure 2:**
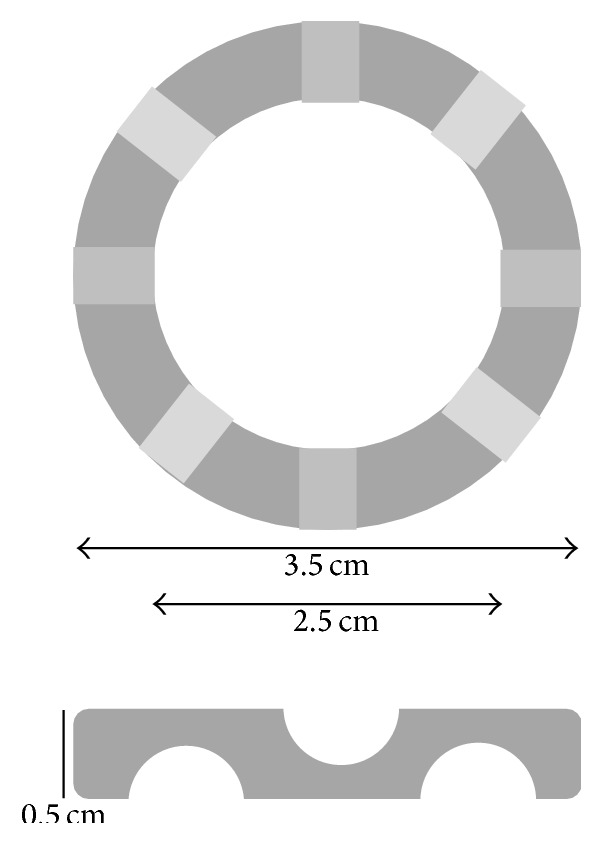
Description of the support device used. This support was previously used by many research teams [2, 4, 6, 8, 9]. It is a plastic ring made with Plexiglas with an outer diameter of 35 mm and an inner diameter of 25 mm. Its height is 5 mm. Eight channels of 2.5 mm of height (depicted in lighter greys) were carved in the plastic (4 in the upper face, 4 in the lower face) at an equal distance.

Cultured Fb at passage three were seeded at confluence (4 × 10^5^ Fb) in 6-well plates including paper anchorage device and cultured in the Fb medium supplemented with 50 *μ*g/mL ascorbate for 14 days. Stromal sheets were detached from the plastic and allowed to lie at the bottom of the well (standard) or flipped on the other side and allowed to lie at the bottom of the well (“flipped”) or elevated on a permeable plastic ring (“stretched”). Then a second seeding took place. Fb were seeded to form a confluent layer on the top of the stromal sheet (4 × 10^5^ Fb). In the case of the stretched condition, culture medium was added three hours later to entirely submerge the sheet. During the seeding step, the addition of the medium containing the Fb creates a light depression at the center of the stromal sheet which is sufficient to maintain medium and cells on it while covering the surface. The paper anchorage is perfectly superimposed on the plastic ring; therefore tissue is not in contact with plastic. Culture was pursued for 14 additional days with Fb medium supplemented with 50 *μ*g/mL ascorbate (Figure 1). Permeable plastic ring is a Plexiglas device described in Figure 2.

### 2.3. Histological Analysis

Sections of each sample were fixed in HistoChoice tissue fixative (Amresco, Solon, OH); then they were cut from the center of the tissue as four rectangles of approximately 4 mm length and 1.5 mm height (exact dimension could vary with the ability of the investigator). After an overnight incubation in HistoChoice, they embedded in paraffin. Histological sections of 5 *μ*m were cut and stained using Masson's trichrome (MT).

### 2.4. Matrix Fibre Angles Determination

Matrix fibre angles versus horizon were assessed using pictures taken with Axio Imager M2 microscope (Carl Zeiss) and analyzed by ImageJ software (NIH, Bethesda, MD) on 25 measurements done on every picture of a MT slide. After opening of the picture file (which could be submitted to a rotation to have a horizontal image of the tissue), a grid of 5 horizontal and 5 vertical lines was applied on the tissue photograph. The angle to the horizon of the part of fibres corresponding to the 25 intersection points generated was measured using the line button and the analysis/measure function. The value was adjusted to fit in a range from 0 to 90 degrees. For example, −160 degrees equals 20 degrees or 175 degrees equals 5 degrees. If there is no fibre at the intersection point, the nearest fibre was chosen. Therefore, there are 25 angle measures per image.

### 2.5. Ratio Length/Width of Cells Embedded in the Stroma

Length/width ratios of cells embedded in the stroma were assessed using pictures taken with Axio Imager M2 microscope (Carl Zeiss) and analyzed by ImageJ software (NIH, Bethesda, MD) on 10 measurements done on 3 different pictures of MT slides. After opening of the picture file, the purple/pink structures corresponding to the cells were identified and associated using the line button with 2 lines making a cross in the center of the presumed nucleus of the cell (when cells seem wavy, several continuous lines could be used). The length of these lines could be measured using the analysis/measure function. The greater measure is associated with the length of the cell and the lower measure with its width.

### 2.6. Thickness Determination

Thickness of stroma was assessed using pictures taken with Axio Imager M2 microscope (Carl Zeiss) and analyzed by ImageJ software (NIH, Bethesda, MD) on 25 measurements done for every 3 MT pictures per reconstructed tissue.

### 2.7. Cell Density Determination

Cell number and cell density were assessed using pictures taken with Axio Imager M2 microscope (Carl Zeiss) by counting cells on 10 slices of reconstructed tissue. Values were normalized to the value obtained for the standard condition.

### 2.8. Statistical Analysis

Values were expressed as mean plus or minus standard error of the mean. Statistical analysis was conducted using Student's *t*-test to compare two data sets. The level of significance was established at *p* < 0.05.

## 3. Results

### 3.1. Organization of Tissues Produced Using the Standard or “Flipped” Techniques Showed Clear Differences Compared to the Tissue Produced Using the “Stretched” Condition

**Figure 3:**
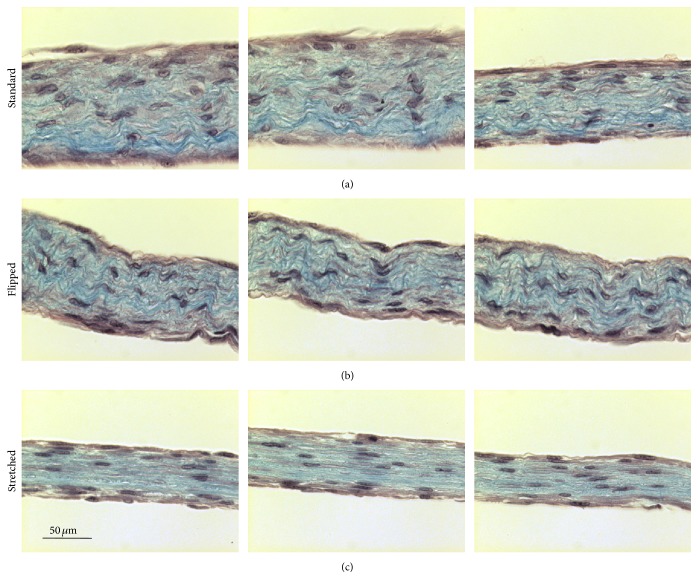
“Stretched” cell culture conditions induced a reorganization of the matrix. Slices of paraffin-embedded tissues produced with standard (a), with “flipped,” or with “stretched” cell culture conditions were subjected to staining using Masson's trichrome staining. Matrix fibres were stained in blue whereas the cells were stained in purple/pink. The bar scale is 50 *μ*m for all photographs. Pictures correspond to 3 representative tissues.

After Masson's trichrome staining, slices of tissue produced using the standard (Figure 3(a)) or the “flipped” (Figure 3(b)) techniques presented a homogenous and compact matrix with a roughly similar distribution of cells. Also, the matrix fibres seem wavy and randomly organized. In contrast, use of the “stretched” technique produced tissues with clear horizontal alignment of matrix fibres and cells, even if the matrix remained compact and cells were homogeneously distributed (Figure 3(c)).

### 3.2. “Stretched” Technique Induced Realignment of Matrix Fibres and Cells in the Engineered Tissue Compared to the Ones Produced by the Standard or the “Flipped” Methods

**Figure 4:**
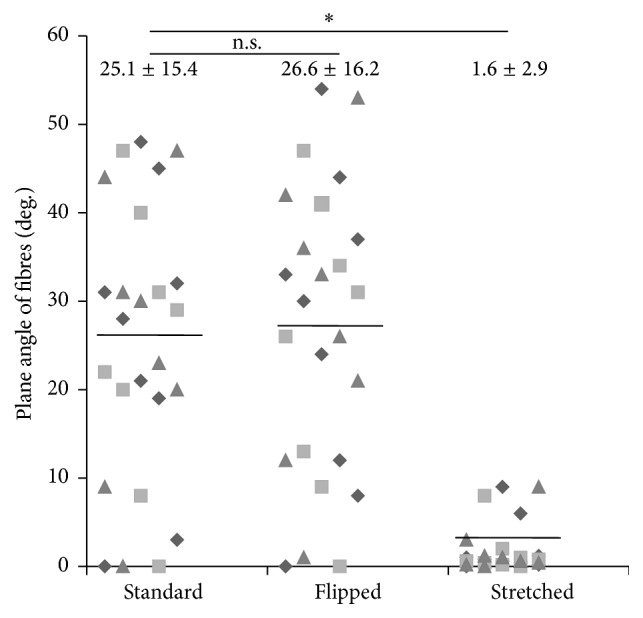
“Stretched” cell culture conditions realigned matrix fibres with the horizon. Masson's trichrome stained slices of tissue were analyzed to determine the plane angle of the matrix fibres. (No fibre was found to have an angle superior to 60 degrees.) Distribution of plane angle of standard, “flipped,” and “stretched” conditions was presented. The bars correspond to the mean also reported with standard deviation on the top of the graph. Triangles, squares, and diamonds represent value of the plane angle of the fibres in the same representative experiment. n.s. is for not statistically significant different compared values and *∗* is for statistically significant different compared values.

Tissues produced by the standard or the flipped techniques produced a matrix where fibres stained by Masson's trichrome technique presented angles varying from 0 to 60 degrees from the horizon. Mean (± standard deviation) of stained fibres angles was 25.1 degrees ± 15.4 and 26.6 ± 16.2 for standard and “flipped” tissues, respectively (Figure 4). No clear orientation or alignment of the fibres or cells in these tissues could be seen contrarily to what was seen when “stretched” technique was used to produce tissues. In these cases, all the fibres presented an angle between 0 and 10 degrees from the horizon (Figure 4). Mean (± standard deviation) of stained fibres angles was 1.68 ± 2.95 for “stretched” tissues (significantly different from standard and “flipped” with *p* < 0.0001).

### 3.3. Cells Embedded in Stroma Were More Flat in the Tissue Produced by the “Stretched” Technique Than the One Using the Standard Protocol

**Figure 5:**
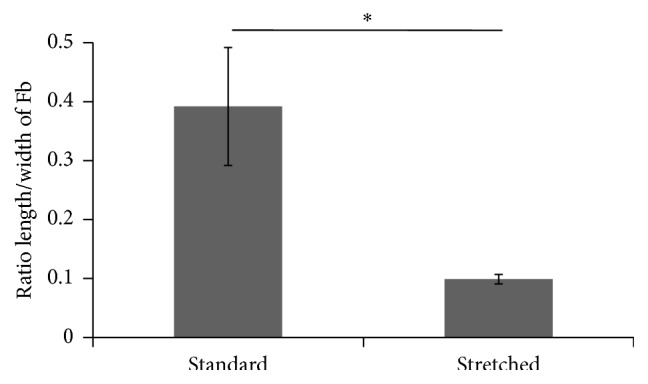
“Stretched” cell culture conditions flattened the cells embedded in the stroma. Masson's trichrome stained slices of tissue were analyzed to determine the ratio between the maximal length and the maximal width of cells. Asterisks indicate when statistically significant differences were found (i.e., *p* < 0.05).

The ratios between the maximal length and the maximal width of the cells included in the reconstructed stromas were analyzed to determine if the orientation of the matrix fibres had an influence on the morphology of the cells. A value of 1 indicates a round cell; a value near 0 indicates a virtually linear cell. Cells from the tissue reconstructed using the standard technique had a ratio of 0.39 ± 0.10 versus 0.10 ± 0.01 for the cells embedded in the stromas produced with the “stretched” protocol,* N* = 3, *n* = 10, *p* value < 0.04 (Figure 5).

### 3.4. Cell Count and Thickness of the Tissue Were Reduced in Tissues Produced Using “Stretched” Technique Compared to Standard and “Flipped” Techniques

**Figure 6:**
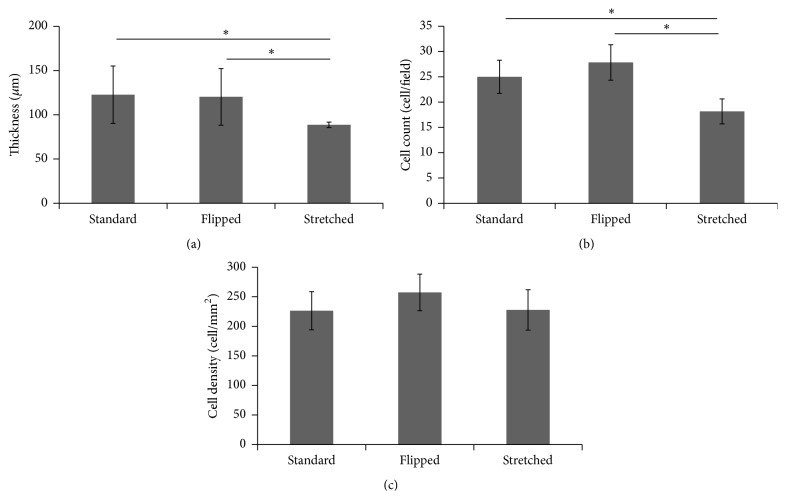
“Stretched” cell culture conditions reduced the thickness of the tissue and the cell count. Asterisks indicate when statistically significant differences were found (i.e., *p* < 0.05). (a) Thickness of the tissue was determined using photographs of slices stained with Masson's trichrome. (b) Cell count was also determined from the same photographs. (c) Cell density was determined by dividing the number of cells counted inside the tissue per area of this tissue.

The thickness of the tissue produced using the “stretched” technique was significantly reduced (−18%) when compared with the two other conditions (Figure 6(a)): 88.6 *μ*m ± 3.1 (“stretched”) versus 122.7 *μ*m ± 32.4 (standard) or 120.2 *μ*m ± 32 (“flipped”), *p* = 0.001. There was no increase in staining seen in the different tissues whatever the technique used. Cell count was also significantly reduced for “stretched” made tissues compared to other techniques (Figure 6(b)): 18.1 ± 2.5 versus 25 ± 3.2 and 27.8 ± 3.5 for standard and “flipped” made tissues, respectively (*p* < 0.0001). Nevertheless, the cell density remained similar for all the tissues produced: 226.4 cells/cm^2^ ± 32.3, 257.3 ± 30.9, and 227.8 ± 34.2 for standard, “flipped,” and “stretched,” respectively.

## 4. Discussion

In a historical perspective, self-assembly technique consists in stacking matrix sheets produced by cultivating fibroblasts in the presence of ascorbate for 4 weeks [49, 50]. This technique is called “sheet stacking self-assembly” in this text. Here, a variant of the “sheet stacking self-assembly” technique, called “reseeding self-assembly,” was used, in which fibroblasts were reseeded after 2 weeks and no stacking step is needed. Cultures were then pursued for 2 additional weeks. The tissues produced using “reseeding self-assembly,” called standard for this study, show a more homogenous distribution of cell throughout the stroma without space delineating the matrix sheets as seen when cell sheets are stacked like in the “sheet stacking self-assembly” technique [51]. The difference between these techniques is important. In the “sheet stacking self-assembly” the cells were mostly found at the bottom of the stromal sheets suggesting they have few contacts with the ECM, while fibroblasts were found throughout the reconstructed tissue when the “reseeding self-assembly” protocol is used, suggesting the cells have more contacts with the ECM.

In several studies, complex techniques to evaluate collagen fibre alignment using staining and observation with polarized light or the two-photon technology combined with the use of fast Fourier transform. We had chosen a lighter kind of analysis due to the obvious difference of the results obtained between our experimental conditions. Because we cannot assume the fibres stained in Masson's trichrome were only collagen, we called them MT stained fibres. Nevertheless, Figure 3 is one excellent illustration of the maxim: an image is worth a thousand words.

In the “sheet stacking self-assembly,” the majority of the mesenchymal cells stay at the bottom of the tissue, near the plastic surface, and matrix is deposited above. This accumulation of cells is responsible for the lines of weakness which separate the stacked sheets [50]. In this study, the first step, before the reseeding, produces the same kind of tissue. Therefore, by flipping the matrix sheet before reseeding dermal fibroblasts on top, we reseeded the cells on the surface of tissue mainly constituted of cells, contrarily to the standard technique where the second layer of fibroblasts is reseeded on a tissue mainly constituted of matrix. Nevertheless, no difference in the parameters, which have been measured in this study, could be observed between tissues produced by these techniques with a similar tissue organization (Figures 3, 4, and 5), tissue thickness (Figure 6(a)), and cell count (Figure 6(b)).

Elevation of the matrix sheet on the permeable ring of plastic induced a mechanical tension and the literature demonstrated that such a tension could cause a realignment of the collagen fibres, an adaptation of the tissue to the mechanical constraints. It was previously demonstrated that reseeding fibroblasts on the top of a matrix sheet induced a transitory peak of matrix metalloproteinase (MMP) [51]. These MMPs could then help to realign the fibres in response to the mechanical stress but such a tension could also induce MMP by itself [52, 53]. Whatever the origin of the MMP or the molecular mechanism of the realignment of the matrix, which could be further investigated, the “stretched” made tissue clearly presents matrix fibres and cells aligned to the horizon compared to the standard and “flipped” conditions (Figures 3 and 4). Previous studies indicate that the cell alignment precedes the matrix fibre alignment [54]; in this work, this aspect was not evaluated, but it could be interesting to investigate this point in the future. It seems that the homogenous orientation of the tissue came with a flattening of the cells (Figure 5) and a lower cell count (Figure 6(b)). As the same number of cells was seeded in each condition, this difference could result from a decrease in cell proliferation or the death of fibroblasts due to the stress or the reorganization of the stroma. It is interesting to note that it was previously reported that, in ageing skin, the cell presents a lower proliferation rate [55, 56].

Stretching could induce proliferation and apoptosis. Valve interstitial fibroblasts react to a cyclic strain by increasing, at the same time, proliferation and apoptosis. Combination of these two opposite biological processes could then result in an increasing number of cells or a reduced one: in this case, cell number decreased [56]. Vascular smooth muscle cell proliferation is attenuated by cyclic strain [57] whereas the proliferation of the endothelial cells, which are constantly submitted to stress, is increased [58]. The c-Jun N-terminal kinase (JNK) was found increased in canine patellar tendon cells, also regularly under tension. A persistent activation of JNK is also known to be proapoptotic, so an overuse of tissue, which places it in a constant stress environment, could be detrimental [59]. Nevertheless the intensity of the stress also plays a key role: osteoblastic cell proliferation in vitro is increased under stimulation with physiologic cyclic strain but is decreased when the stimulation was in a supraphysiological range [60]. In cancer, the tension on the tumour tissue, that is, compressive stress, from the surrounding ECM, could contribute to cell growth regulation with more apoptosis and less proliferation in high-stress area and less apoptosis and more proliferation in low stress area [61]. In the present study, we could note that the cell number was decreased under “stretched” conditions but proliferation and apoptosis were not evaluated. In the same way, the intensity of the stretching was not measured. Experiments could be done in the future to further characterize the technique.

The “stretched” made tissue presented a thinner stroma. This thinning probably did not result from the compaction of the tissue because the MT staining was not more intense. The reduced number of cells in the tissue could nevertheless explain a lower production of ECM molecules and then a thinner stromal layer. Further studies, such as collagen deposition kinetics, were needed to elucidate this phenomenon.

The “stretched” technique is a simple technique which could be used to study ageing and cancer. Interestingly, skin of mice had a collagen orientation index of 0.32 at 8 weeks when this value was 0.78 at 60 weeks. Random orientation has a 0 value, when 1 is perfect parallel tissue. These results indicate a profound remodelling of ECM and an alignment of the matrix fibres during the ageing [25]. It will be interesting to investigate if an epidermis reconstructed on ageing stroma explants versus a “stretched” stroma shows the same features.

In the same way experiments using healthy, precancerous, and cancer cells on stromas produced with standard or “stretched” techniques could be compared with behaviour described in literature for such cells.

Scars often happen after a problematic wound healing especially when tensile forces of the skin were broken. Some regions of the body are more prone to produce this inadequate form of healing: anterior chest, for example, produces scars and keloids. Strong differences exist between the regions of the body in terms of vertical and horizontal alignment of collagen fibres [62]. Corneal stroma is another tissue where alignment of collagen fibres is essential and where scarring could be problematic. The control of scar tissue formation is required to keep a clear vision [63]. “Stretched” tissue could be useful tools for in vitro studies of consequences of incisions into dermis or corneal stroma.

## 5. Conclusions

Organization of the matrix fibres is the most important result of this study. The culture of the stromal sheet on a permeable ring in the “stretched” technique allows the total reorganization of the tissue in the 15 days following the second seeding of fibroblasts. In this study neither chemical treatment/micropatterning of the plastic nor use of exogenous material was required. The permeable ring is not integrated in the tissue and is reusable. This family of self-assembly techniques are relatively inexpensive with a cost of such a tissue inferior to 1$ per centimetre square, making it accessible to all the research laboratories.
